# Effects of Basal Selenium Fertilizer Application on Agronomic Traits, Yield, Quality, and Se Content of Dryland Maize

**DOI:** 10.3390/plants11223099

**Published:** 2022-11-15

**Authors:** Le Wang, Fei Gao, Liguang Zhang, Li Zhao, Yan Deng, Hongxia Guo, Lixia Qin, Chuangyun Wang

**Affiliations:** College of Agronomy, Shanxi Research Institute of Functional Agriculture, Shanxi Agricultural University, Taiyuan 030031, China

**Keywords:** agronomic trait, maize, quality, selenium content, selenium fertilizer

## Abstract

To explore the efficiency of selenium (Se) fertilizer application in dryland maize, we tested five Se fertilizer application treatments: 0 g ha^−1^ (Se0), 75 g ha^−1^ (Se1), 150 g ha^−1^ (Se2), 225 g ha^−1^ (Se3), and 300 g ha^−1^ (Se4). Compared with Se0, Se2 increased the leaf area, chlorophyll content, internode length, plant height, and ear height by 7.95%, 3.20%, 13.19%, 1.89%, and 7.98%, respectively. Se2 and Se3 significantly affected the stem internode diameter, cortex thickness, and cellulose content, which were positively correlated with lodging resistance. Compared with Se0, Se3 promoted the contents of soluble sugar, crude protein, crude fat, and starch in grains, which increased by 9.48%, 6.59%, 1.56%, and 4.82%, respectively. It implies that maize grain application of Se significantly improves their Se content. Se1 did not observably influence the growth of maize, and the promoting effect of Se4 on maize decreased. The lodging resistance of maize as analyzed by Pearson correlation analysis correlated with the application of Se fertilizer. It proved that higher yield, grain quality, grain Se content, and lodging resistance of stems were concerned with Se fertilizer application in the range of 150–225 g ha^−1^. The results provide useful information for Se fertilizer treatment in dryland maize.

## 1. Introduction

Maize (*Zea mays* L.) is the largest food crop in China, and it is the third major cereal crop throughout the world [1]. In terms of the human diet, maize provides at least 30% of food calories to more than 4.5 billion people, especially in developing countries [2]. Maize is high in starch and rich in antioxidant compounds such as phenolic compounds, carotenoids, and lutein [3]. Food is the main source of human nutrition, and the food ingested should provide all the necessary nutrients for the human body. As a result of the success of the Green Revolution, more high-yield cereal crops were planted by farmers, leading to increased feed and food production. However, this trend has led to a decrease in micronutrient concentrations in food and feed and inadvertently exacerbates the problem of hidden hunger [4]. In China, surface soils are generally Se-deficient, with an estimated 51% of China below or near the deficiency level (0.125 mg kg^−1^) [5] and with nearly 15% of the global population [6]. To improve the lack of micronutrients in food production on poor soils, agricultural practices such as the application the micronutrient fertilizer have been developed. 

Drought stress is one of the major constraints on global agricultural productivity [7]. The repeated occurrence of drought makes plants more susceptible to the effects of water shortage, which inhibits the physiological and metabolic processes of plants, affects the growth and development of plants, and seriously limits yield and productivity [2]. For example, photosynthesis, one of the main metabolic processes that affects crop yield, is directly affected by drought [8]. The arid and semi-arid regions of the world, especially in developing countries, are at great risk as they face more severe water shortages [9]. The challenge is to develop better drought resistance strategies to increase dryland maize yields under limited water resources. 

Se is an essential trace mineral that plays an important role in human functioning, including in Se antioxidant proteins [10]. Se is present as an essential micronutrient in the form of selenoprotein, Se cysteine, and selenomethionine (SeMet), which affect important biological functions such as free radical metabolism, immune function, and apoptosis [11]. 

Se is not considered essential in plants, in contrast to its requirement in humans and animals [12]. Studies have shown that application of Se fertilizer can promote plant growth [13], and the appropriate Se concentration has a certain role in promoting plant production [14]. The application of exogenous Se can increase chlorophyll and carotenoid content in plant leaves [15] and enhance the photosynthetic capacity of plants. What is more, Se could improve the yield of maize [16], rice [17], and increase the Se content and nutritional value of crops [1,18]. 

In addition, Se fertilizer application is an effective auxiliary means for plants to combat drought stress [1]. However, Se improves tolerance to drought stress by regulating the water status of plants [19]. For example, the application of Se fertilizer can alleviate the adverse effects of drought stress on the growth of rice [20]. 

Maize is widely cultivated around the world and is an important food crop, so maize is essential for dietary Se intake [21,22]. The worsening weather has a bad effect on the growth and development of maize. Adding fertilizer is a convenient, effective and low-cost way to improve plant tolerance to stress. Due to the application of fertilizer being affected by different climatic conditions, Se application may vary from place to place [23]. For example, the water content in the soil affects the absorption of Se fertilizer applied to maize plants [24]. In addition, high Se concentration is harmful to plants, leading to serious physiological disorders [25], and low Se concentration has no effect on plant growth. Therefore, determining the appropriate application range of Se fertilizer needs to be solved as soon as possible. Under field conditions, this study aimed to explore the effects of basal Se fertilizer on agronomic traits, grain quality, and yield of dryland maize; select the appropriate application range for promoting maize growth; and suggest a scientific basis for further development of productive Se-enriched dryland maize. 

## 2. Results

### 2.1. Maize Yield

The grain yield was under the significant effect of Se fertilizer application (Table 1). The grain yield was more powerful with the change of Se fertilizer application at location SY in comparison to location DY. At location SY, Se influenced the grain yield significantly, and Se1, Se2, Se3, and Se4 increased by 8.26%, 12.25%, 7.90%, and −5.81%, respectively, and the grain yield was the highest in the Se3. The number of ears of maize was also affected by the amount of Se fertilizer applied, and compared with the Se0 treatment, the Se1, Se2, Se3, and Se4 treatments increased by 8.98%, 9.40%, 8.59%, and 0.86%, respectively. At location SY, the Se2 was significantly higher than the Se1, Se3, and Se4, with no significant difference between the Se1 and Se3. The application of Se fertilizer increased the grain’s number and grain yield, which showed a trend of first increasing and then decreasing after application. Thus, the maximum record values of the grain number and grain yield were observed in Se2.

### 2.2. Dry Weight of the Plant

With the increase in Se application, the dry weight of plants showed significant differences between treatments (Table 2). At location DY, the dry weight of stems, leaves, ears, maize cobs, and grains were increased by 12.61%, 10.78%, 14.67%, 5.03%, and 16.06%, respectively, in Se1 compared with Se0. At location SY, compared with the Se0, the dry weight of stems, leaves, ears, maize cobs, and grains were significantly influenced by Se, which in the Se1 were increased by 25.46%, 11.19%, 21.98%, 24.12%, and 21.59% respectively; the Se2 were increased by 35.07%, 14.26%, 33.14%, 32.46%, and 33.22%, respectively; and the Se3 were increased by 16.19%, 7.66%, 26.35%, 19.86%, and 27.32%, respectively. The total weight showed a non-significant increase with the Se application. The Se1, Se2, and Se3 increased the dry weight of stems, leaves, ears, maize cobs, and grains, and the improvement effect was the best in Se2. 

### 2.3. Leaf area and Chlorophyll Content

In the tasseling period, the leaf area of DY Se1, Se2, Se3, and Se4 were increased by 13.92%, 32.32%, 33.17%, and 33.29%, respectively compared with Se0. The leaf area of SY for the Se1 and Se2 were increased by 1.16% and 3.11%, respectively compared with Se0, but Se3 and Se4 showed a non-significant effect on leaf area (Table 3). In the milk stage, at location DY, the leaf area of Se1, Se2, Se3, and Se4 increased by 11.37%, 5.08%, 2.39%, and 3.81%, respectively compared with Se0, and there was no obvious difference at location SY. In the bighorn mouth stage, the chlorophyll in DY Se1, Se2, Se3, and Se4 increased by 1.39%, 5.69%, 4.29%, and 0.34%, respectively, compared with Se0. The chlorophyll of SY in the Se1, Se2, Se3, and Se4 treatments increased by 7.01%, 3.43%, 7.05%, and 0.41%, respectively, compared with Se0 in the milk stage. Pearson correlation analysis showed leaf area tasseling was significantly positively correlated with Se fertilizer (Table 4 and Table 5). 

### 2.4. Internodal Length, Plant Height, and Panicle Height

With the gradual increase of Se application, the internodal length tended to decrease first and then increase as a whole (Table 6). The length of the third, fourth, and fifth stems of maize in the milk stage was minimized at the Se3 treatment. The third stem sections in Se1, Se2, Se3, and Se4 decreased by 14.13%, 13.19%, 21.89%, and 9.99%, respectively compared with Se0, and the fourth stem section in Se1, Se2, Se3, and Se4 decreased by 9.19%, 8.39%, 18.00%, and 11.45%, respectively compared with the Se0. For the fifth stem section, Se1, Se2, Se3, and Se4 were treated with a decrease of 9.33%, 10.21%, 17.56%, and 12.12%, respectively, compared with the Se0. Plant height and panicle height increased first and then decreased with the increase of Se application. The plant height of Se1, Se2, Se3, and Se4 decreased by 0.89%, 0.71%, 1.51%, and 1.33%, respectively compared with Se0. The difference in Se fertilizer treatment in the milk stage was not significant; the ear height of Se1, Se2, Se3, and Se4 decreased by 1.54%, 7.98%, 6.29%, and 1.54% respectively compared with Se0 treatment.

### 2.5. Stem Strength and Internodal Diameter

**Figure 1:**
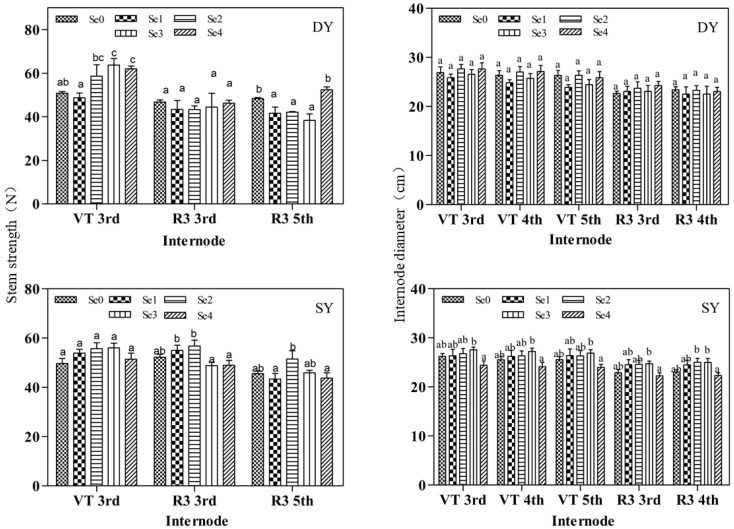
Effects of Se fertilizer application on stem strength and internode diameter of maize at locations DY and SY. Note: Se0, Se fertilizer application 0 g ha^−1^; Se1, Se fertilizer application 75 g ha^−1^; Se2, Se fertilizer application 150 g ha^−1^; Se3, Se fertilizer application 225 g ha^−1^; Se4, Se fertilizer application 300 g ha^−1^. Different lowercase letters in the same column indicate significant differences among different treatments (*p* < 0.05).

Se fertilizer has a significant effect on the stem strength and internodal diameter of maize (Figure 1). Pearson correlation analysis showed the stem strength tasseling third was significantly positively correlated with Se fertilizer (Table 4 and Table 5). During the tasseling period, the stem strength of the DY was significantly different during the Se2, Se3, and Se4, which were increased by 15.41%, 25.45%, and 22.05%, respectively, compared with Se0. There was no significant difference in the stem strength of the SY, but the stem strength in Se2 and Se3 was larger, at 12.12% and 12.72% higher than that of Se0, respectively. The diameter of the internodes showed a trend of increasing first and then decreasing with the increase of Se application, with the most significant under the Se3, the third, fourth, and fifth stem nodes of the male, the third and fourth stem nodes of the milk ripe increased by 5.11%, 6.70%, 5.25%, 7.96%, and 8.55% respectively, compared with Se0. 

### 2.6. Cortex of Maize Stalk

**Figure 2:**
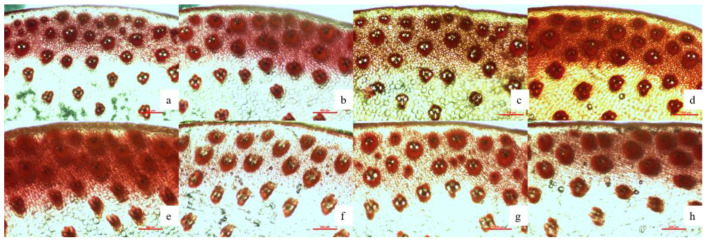
The Structure of the center vascular bundle of the 3rd basal internodes of maize treated with Se fertilizer. Note: (**a**–**d**) represent dongyang Se0, Se2, Se3 and Se4,respectively (×40); (**e**–**h**) represent shouyang Se0, Se2, Se3 and Se4,respectively (×40). Se0, Se fertilizer application 0 g ha^−1^; Se2, Se fertilizer application 150 g ha^−1^; Se3, Se fertilizer application 225 g ha^−1^; Se4, Se fertilizer application 300 g ha^−1^. Different lowercase letters in the same column indicate significant differences among different treatments (*p* < 0.05).

The thickness of the tissue at the base of the third stem varied significantly with the change of Se application (Figure 2). The result of Pearson correlation analysis showed that hard tissue thickness and crude fiber were significantly positively correlated with Se fertilizer (Table 4 and Table 5). The maximum value was reached under Se3 in the DY, and the maximum value under Se2 was reached in the SY. Compared with the Se0, the Se2, Se3, and Se4 increased by 5.74%, 9.02%, and 5.74%, respectively at location DY (Table 7); the Se2 and Se3 treatments increased by 4.00% and 0.80%, respectively, at location SY. The crude fiber of the Se2, Se3, and Se4 increased by 8.41%, 11.30%, and 11.01%, respectively, compared with the Se0 at location DY. At location SY, the Se2 and Se4 increased by 1.57% and 13.35%, respectively, compared with the Se0. 

### 2.7. Maize Quality

At location DY, the soluble sugar content first increased and then decreased with the Se treatment (Table 8). The Se1, Se2, and Se3 increased by 4.78%, 5.22%, and 3.04%, respectively, compared with the Se0. The starch and soluble sugar content changed similarly. Treatment Se1, Se2, Se3, and Se4 increased by 0.43%, 2.99%, 3.70%, and 3.13%, respectively, compared with the Se0. At location SY, the crude protein content also presented a trend of rising and reducing with the treatment of Se, with the Se1, Se2, Se3, and Se4 increased by 4.30%, 7.34%, 1.65%, and 1.01%, respectively, compared with the Se0. The crude fat content showed a similar trend with the crude protein, with Se1, Se2, Se3, and Se4 increased by 13.79%, 10.34%, 3.45%, and 3.45%, respectively, compared with the Se0. The starch content and soluble sugar content were similar, and the starch content reached a peak in the Se4 treatment, an increase of 7.33% compared with Se0, and the soluble sugar content reached a peak in the Se3, an increase of 15.29% over Se0. The result of Pearson correlation analysis showed that soluble sugar, crude protein, and starch were significantly positively correlated with Se fertilizer (Table 4 and Table 5). 

### 2.8. Se Content in Maize Grains

**Figure 3:**
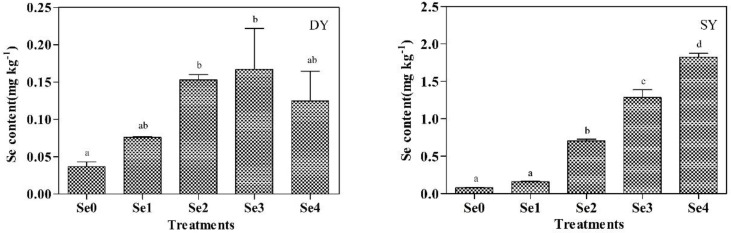
Effects of Se fertilizer application on the Se content in maize grains at locations DY and SY. Note: Se0, Se fertilizer application 0 g ha^−1^; Se1, Se fertilizer application 75 g ha^−1^; Se2, Se fertilizer application 150 g ha^−1^; Se3, Se fertilizer application 225 g ha^−1^; Se4, Se fertilizer application 300 g ha^−1^. Different lowercase letters in the same column indicate significant differences among different treatments (*p* < 0.05).

The Se content in maize grains was increased with the Se fertilizer application (Figure 3). The result of Pearson correlation analysis showed that Se content and Se fertilizer had a significant positive correlation in the DY and SY (r = 0.579, 0.963, respectively) (Table 4 and Table 5). The grain Se content of the DY was significantly different during the Se1, Se2, Se3, and Se4, which were increased by 106.59%, 316.25%, 354.31%, and 239.21%, respectively, compared with Se0. Compared with the Se0, the Se1, Se2, Se3, and Se4 increased by 107.71%, 812.52%, 1563.29%, and 2264.07%, respectively, at location SY. The maximum value was reached under Se3 in the DY, and the maximum value under Se4 was reached in the SY. What is more, the Se content in maize grains of the SY was higher than that of DY.

## 3. Discussion

### 3.1. Effects of Basal Se fertilization on Maize Yield and Quality

Previous studies have shown that the effect of Se fertilizer application on the growth of maize is not a single linear relationship, and the appropriate amount of Se fertilizer is beneficial to the growth of plants [26], with the overall trend of first rising and then declining with the increase of Se fertilizer application. Drought was also a dominant factor limiting yields, and Se can maintain osmotic pressure and increase photosynthetic pigments, which helps plants produce more biomass under arid conditions [27]. The application of Se fertilizer to dryland maize was beneficial to increase its drought tolerance and regulate its growth. Se, by improving the antioxidant activity of plants, thereby increases yields. However, the Se fertilizer had divergent effects on crops; for instance, there were no significant effects on wheat yield [28], which may be related to the different species, the Se fertilizer type, and the method of fertilization. The application of exogenous Se in the soil activates soil microorganisms and promotes their activity [29]. Se fertilizer was applied to the soil before sowing in this study, and Se had a positive effect on the entire growing period of the plant. The results of this study showed Se fertilizer application in the range of 150–225 g ha^−1^ can increase maize yield by 7.90–12.25%, the number of ears of maize by 8.59–9.40%, and improve the quality of maize grains, which was similar to the previous findings on oats [14]. At location DY, the soluble sugar content and starch content in the Se3 treatment increased by 15.29% and 5.92%, respectively. Studies have shown that the application of Se fertilizer can increase the protein content of maize [27], fragrant rice [17], grapes [30], and other crops, which is consistent with our results. 

### 3.2. Effects of Se Fertilization on Agronomic Traits of Maize

Drought affects the physiological traits of maize and inhibits its growth [31]. Se fertilizer application can maintain plant water status, regulate the content of osmotic regulatory substances [7], enhance antioxidant activity [1], increase pigment content, and regulate photosynthesis [32]. Therefore, the application of Se fertilizer can enhance the tolerance of plants to drought stress and promote the growth of dryland maize. The application of Se fertilizer can regulate the biosynthesis of porphyrin in plants. Chlorophyll formation is associated with porphyrin, so Se fertilizers can promote the formation of chlorophyll [33]. Se improves photosynthesis by influencing the synthesis of plant chlorophyll and regulating electron transport during photosynthesis, thereby increasing the chlorophyll content and photosynthetic capacity in plant leaves. Studies have shown that applying Se fertilizer can increase the chlorophyll content in maize [2], fragrant rice [17], and Lycium chinense [15]. In addition, due to the complexity of chlorophyll biosynthesis and the involvement of many elements, it is possible that the increased chlorophyll content caused by Se application facilitates the uptake of mineral elements involved in chlorophyll biosynthesis [15,34]. This study shows that with the continuous improvement of Se application, the leaf area size and chlorophyll content show a trend of increasing and then decreasing but without significant differences between different treatments (Table 3). The 150–225 g ha^−1^ Se application range can increase the chlorophyll content by 3.43–7.05% and promote the photosynthesis of crop leaves. Plants grown in soil fertilized by adding 50 mg kg^−1^ of Se can promote chlorophyll synthesis and promote crop metabolism [2]. Appropriate application of fertilizer could improve the growth and delay the senescence of dryland maize. However, the specific effect requires further research. 

### 3.3. Effects of Se Fertilization on Maize Stalks

This study shows that with the increase in Se fertilizer application, the internode diameter and cortex thickness (Table 7) of maize show a trend of first increasing and then decreasing, and the Se2–Se3 range is the best. Luo et al. [35], by treating rice subjected to drought stress with Se, confirmed that Se application could increase the stem thickness of rice. The administration of exogenous Se can improve the anatomical characteristics of wheat stems, increasing the diameter of the stem, the thickness of the stem tissue, and the diameter of the vascular bundle [36]. In this study, it was demonstrated that the application of Se fertilizer increased the cellulose content of maize stalks. Maize internodal diameter, stem tissue thickness, and stem cellulose content correlate with the stem strength of maize plants [37]. The lodging resistance of maize stalks is crucial in the growth of maize, and the pitching of stalks at different periods can seriously affect maize growth, yield, quality, and mechanized harvesting. Increasing the stem strength of maize plants and reducing lodging in planting by applying appropriate amounts of Se fertilizer is of great practical significance for the planting of dryland maize.

### 3.4. Effects of Basal Se Fertilization on Se Content in Maize Grains

The Se content in maize grains was more important in maize enriched with Se. The Se fertilizer application increase could improve the Se content in grains significantly [38]. With Se fertilizer application, the grain’s Se content in DY increased by 106.59–316.25% (Figure 3). Soil Se application significantly increased the Se content of plants [2]. The Se fertilizer applied to the soil is transferred from the underground to the aboveground through the roots and stems, where it is absorbed, operated, and transformed into the different tissues of maize [39]. The Se contents of the fundamental fertility in the SY were significantly higher than DY. The Se content in the plant was consistent with the change in soil Se content [1]. In addition, there was also a difference in the precipitation levels at locations DY and SY. The water regimes of the soil showed a different effect on maize enriched with Se [1]. Our study shows different Se fertilizer application has a significant effect on the Se content of maize grains, and the results provide a scientific basis for the Se-rich cultivation of dryland maize. The same fertilization treatment was carried out in both regions, but the impact was inconsistent. We will analyze other factors such as soil fertility and precipitation in subsequent tests to further study the specific mechanisms.

## 4. Material and Methods

### 4.1. Site Description and Experimental Design

The experiment was during May to October 2021 in Dongyang Experimental Base of Shanxi Agricultural University (DY), China (37°11′ N, 113°06′ E) and Shanxi Shouyang Maize Science and Technology Institute Experimental Base (SY), China (37°55′ N, 112°68′ E). The average annual temperature in DY is 9.8 °C and the average annual rainfall is 422.2 mm, which is mainly concentrated in July–September. The experimental soil is of yellow clay type. The basic physicochemical properties of the 0–20 cm soil layer are as follows: 13.06 g kg^−1^ organic matter, 0.93 g kg^−1^ total nitrogen, 17.08 g kg^−1^ total potassium, 0.754 g kg^−1^ total phosphorus, 66.5 mg kg^−1^ alkaline nitrogen, 10.9 mg kg^−1^ available phosphorus, 109 mg kg^−1^ available potassium, a pH of 8.49, and 0.290 mg kg^−1^ Se. The average annual temperature in SY is 7.6 °C, the average annual rainfall is 500 mm, and the precipitation is mainly concentrated in July–September. The experimental soil is brown type. The basic physicochemical properties of the 0–20 cm soil layer are as follows: 16.21 g kg^−1^ organic matter, 1.15 g kg^−1^ total nitrogen, 19.66 g kg^−1^ total potassium, 1.248 g kg^−1^ total phosphorus, 17.5 mg kg^−1^ alkaline nitrogen, 7.4 mg kg^−1^ available phosphorus, 74 mg kg^−1^ available potassium, a pH of 8.57, and 0.324 mg kg^−1^ Se. 

The seeds of spring maize (*Zea mays* L.) hybrid “Dafeng 30” were provided by Dafeng seed industry. The Se-enriched organic fertilizer (Se content 1 g kg^−1^, NPK nutrient content ≥ 5%, organic matter content ≥ 45%) was produced by Suzhou Se Valley Technology Co., Ltd., Suzhou, China. 

Five Se fertilizer application treatments were tested: 0 g ha^−1^ (Se0), 75 g ha^−1^ (Se1), 150 g ha^−1^ (Se2), 225 g ha^−1^ (Se3), and 300 g ha^−1^ (Se4), and randomized complete blocks were designed with repetition three times, involving a total of 30 plots. Se fertilizer was applied to the soil before sowing in the two planting areas of DY and SY. Each plot was 40 m^2^ (4 m × 10 m) with a planting density of 75,000 ha^−1^. Field management measures, including fertilizing, irrigating, and weeding, were taken from the local high-yield field management method and were managed following the planting method of rainfed areas. 

### 4.2. Agronomic Trait 

During the period of male extraction and milk ripening, the leaf area, internode length, plant height, and ear height were determined according to conventional methods. The stem strength was measured by using a stem strength tester (YYD-1, Zhejiang Top Instrument Co., Ltd., Hangzhou, China). The internode diameter was measured using a vernier caliper with a minimum scale of 0.01 mm.

The SPAD values was correlation with chlorophyll, which can reflect the level of chlorophyll content of the crop [40]. The chlorophyll contents were calculated employing a chlorophyll meter (SPAD-502Plus, Konica Minolta Investment Ltd., Tokyo, Japan), which has been used by many scholars to obtain plant chlorophyll content data [41]. During large trumpet mouth, male extraction, and milk ripening, the SPAD values were measured at the top, middle, and bottom of the ear leaf, and the average values of the three parts were recorded. SPAD values (X) were converted to chlorophyll content (in μmol m^−2^): chlorophyll content = 0.375 X^2^ + 6.630 X + 71.554 [42]. 

Three representative plants were selected from each plot to measure the agronomic traits of maize. 

### 4.3. Plant and Soil Collection and Processing

Samples were obtained during the period of maturity, and six plants were randomly selected within each plot. Three were divided into stems, leaves, and ears and then encapsulated in kraft paper bags. Next, the plants were dried in the oven at a constant 80 °C to analyze their dry weight; the stem and grains were ground into a powder with an electric grinder (HC-200, Zhejiang Yongkang Jinsui Machinery Factory, Jinhua, China) and sealed in paper bags for analysis of the crude fiber of the stem and the contents of soluble sugar, crude protein, ether extract, and starch in grains. The third internode from the bottom of the stem was sampled and soaked in Kano fixative solution, then preserved with 70% ethanol for observing stem microstructure. 

### 4.4. Plant Analyses

Conventional methods determined the total spike number, yield, and 1000-grain weight. The crude fat, crude protein, and crude fiber of maize grains were determined according to the methods described by [43]. 

About 1.5 cm was selected from the middle of the third internode from the bottom of the stem and then stained with 0.1% Ferro red (Tianda Chemical Reagent Factory, Tianjin, China). A microscope (Nikon Ni-U, Tokyo, Japan) was used to observe the vascular bundle structure of the stem, and the thickness of the cortex and hard tissue was measured with a microscale. 

The maize flour (0.3 g) was microwave digested (MARS6 One-Touch, American CEM Corporation, America) with a mixture of HNO_3_-H_2_O_2_ (*v*/*v* ratio of 3:1), according to the method in the references [2,38]. Then, using 1000 mg L^−1^ standard liquid Se as the standard reference materials and 5% hydrochloric acid as the carrier, the Se contents in the digestion solution were measured by hydride generation-atomic fluorescence spectroscopy (AFS-9750, Beijing Haiguang Instrument Co. Ltd., Beijing, China, the limits of detection was 0.01 μg L^−1^), with the Chinese National Standards GB5009.93-2017 (the limit of detection was 2 μg L^−1^ and the limit of quantification was 6 μg L^−1^).

### 4.5. Statistical Analysis

All data were analyzed with SPSS 16.0 (SPSS, Inc., Chicago, IL, USA) to examine differences. Statistically significant differences (*p* < 0.05) were identified using analysis of variance (ANOVA) and least significant difference (LSD) calculations, and GraphPad Prism 5 was used to make the figures.

## 5. Conclusions

Our study showed that when the amount of Se is applied in the range of 150–225 g ha^−1^, it can improve the physiological properties to promote growth and also has a certain promotion effect on the lodging resistance of maize plants and improves the yield, quality, and Se content of grains. Hence, this is the most suitable range of Se application when applying Se fertilizer to dryland maize, and this study provides a theoretical basis for Se fertilizer application in dryland maize. 

## Figures and Tables

**Table 1:** Effects of Se fertilizer application on maize yield at locations DY and SY.

Site	Treatment	Kernels No. Ear^−1^	1000-Kernel Weight (g)	Grain Yield Per Mu (kg)	Grain Yield Per Hectare (kg)
DY	Se0	612.6 ± 69.1ab	316.3 ± 19.5b	980.3 ± 121.0b	14,704.4 ± 1815.1b
	Se1	604.3 ± 28.4ab	290.0 ± 19.6a	881.9 ± 35.9a	13,228.2 ± 537.7ab
	Se2	635.8 ± 44.2b	305.8 ± 18.1ab	979.9 ± 51.0b	14,698.1 ± 765.2ab
	Se3	610.9 ± 43.0ab	289.0 ± 15.2a	878.3 ± 43.8a	13,174.0 ± 656.7a
	Se4	581.0 ± 70.2a	314.5 ± 32.6b	927.5 ± 139.6ab	13,911.8 ± 2093.9b
SY	Se0	640.4 ± 42.2a	293.1 ± 8.5bc	939.2 ± 27.1b	14,087.3 ± 406.3b
	Se1	697.9 ± 49.2b	291.0 ± 6.7b	1016.9 ± 23.4c	15,253.1 ± 350.3c
	Se2	700.6 ± 48.0b	300.3 ± 5.0c	1054.2 ± 17.7d	15,813.2 ± 265.5d
	Se3	695.4 ± 45.1b	290.8 ± 12.8b	1013.4 ± 44.6c	15,200.8 ± 668.5c
	Se4	634.9 ± 42.0a	279.8 ± 7.3a	884.6 ± 23.2a	13,268.2 ± 347.9a

Note: Se0, Se fertilizer application 0 g ha^−1^; Se1, Se fertilizer application 75 g ha^−1^; Se2, Se fertilizer application 150 g ha^−1^; Se3, Se application 225 g ha^−1^; Se4, Se fertilizer application 300 g ha^−1^. Different lowercase letters in the same column indicate significant differences among different treatments (*p* < 0.05).

**Table 2:** Effects of Se fertilizer application on the dry weight of maize at locations DY and SY.

Site	Treatment	Stem (g)	Leaf (g)	Ear (g)	Maize Cob (g)	Grain (g)	The Total Weight (g)
DY	Se0	79.8 ± 8.7ab	29.1 ± 1.2a	192.1 ± 7.0ab	23.8 ± 1.2a	168.2 ± 6.1ab	301.1 ± 15.2a
	Se1	89.9 ± 12.3b	32.3 ± 6.3a	220.3 ± 19.8b	25.0 ± 2.3b	195.2 ± 18.0b	342.0 ± 38.0a
	Se2	83.7 ± 7.4ab	26.8 ± 3.0a	189.3 ± 18.2ab	22.6 ± 1.3ab	166.7 ± 17.0ab	299.8 ± 19.7a
	Se3	64.5 ± 8.7a	22.1 ± 1.4a	154.2 ± 24.9a	18.6 ± 3.5a	135.6 ± 21.4a	240.8 ± 34.6a
	Se4	83.4 ± 18.7ab	27.4 ± 9.0a	187.0 ± 33.9ab	20.9 ± 3.5ab	166.2 ± 30.4ab	297.8 ± 56.6a
SY	Se0	98.4 ± 1.1a	42.3 ± 0.6ab	190.9 ± 9.9a	24.8 ± 1.1a	166.1 ± 9.9a	331.5 ± 10.6a
	Se1	123.4 ± 18.0b	47.0 ± 5.3b	232.8 ± 13.8b	30.8 ± 2.2b	201.9 ± 11.7b	403.3 ± 36.8a
	Se2	132.8 ± 18.3b	48.3 ± 6.8b	254.1 ± 20.8b	32.9 ± 2.9b	221.2 ± 17.9b	435.3 ± 45.0a
	Se3	114.3 ± 7.1ab	45.5 ± 3.3b	241.2 ± 14.1b	29.8 ± 2.6b	211.4 ± 12.0b	401.0 ± 24.4a
	Se4	92.0 ± 10.6a	36.2 ± 3.6a	177.0 ± 22.1a	22.6 ± 2.4a	154.4 ± 19.7a	305.1 ± 35.1a

Note: Se0, Se fertilizer application 0 g ha^−1^; Se1, Se fertilizer application 75 g ha^−1^; Se2, Se fertilizer application 150 g ha^−1^; Se3, Se fertilizer application 225 g ha^−1^; Se4, Se fertilizer application 300 g ha^−1^. Different lowercase letters in the same column indicate significant differences among different treatments (*p* < 0.05).

**Table 3:** Effects of Se fertilizer application on leaf area and chlorophyll content of maize at locations DY and SY.

Site	Treatment	Leaf Area (cm^2^ Plant^−1^)		Chlorophyll Content (μmol m^−2^)		
		Tasseling	Milk Stage	Bighorn Mouth Stage	Tasseling	Milk Stage
DY	Se0	457.2 ± 100.2a	659.7 ± 62.7a	1632.8 ± 84.2a	1827.7 ± 187.7a	/
	Se1	520.8 ± 39.8a	734.6 ± 67.5a	1655.5 ± 76.4a	1764.2 ± 175.7a	/
	Se2	604.9 ± 167.8a	693.2 ± 20.5a	1725.7 ± 244.0a	1845.5 ± 49.2a	/
	Se3	608.8 ± 237.3a	675.4 ± 45.3a	1702.9 ± 124.8a	1824.8 ± 155.1a	/
	Se4	609.4 ± 111.0a	684.7 ± 94.3a	1638.3 ± 76.1a	1797.9 ± 120.6a	/
SY	Se0	701.9 ± 47.5a	748.7 ± 41.8a	1708.6 ± 143.2a	1871.5 ± 153.0a	1543.5 ± 96.6a
	Se1	710.0 ± 65.9a	780.6 ± 38.5a	1752.1 ± 165.3a	1807.5 ± 272.7a	1651.7 ± 269.2a
	Se2	723.8 ± 56.8a	680.3 ± 111.1a	1743.1 ± 96.7a	1814.5 ± 220.4a	1596.5 ± 143.2a
	Se3	684.3 ± 63.8a	722.0 ± 6.4a	1663.2 ± 145.5a	1829.4 ± 190.9a	1652.5 ± 110.6a
	Se4	686.9 ± 64.3a	782.8 ± 85.6a	1637.0 ± 122.1a	1813.0 ± 111.7a	1549.9 ± 193.8a

Note: Se0, Se fertilizer application 0 g ha^−1^; Se1, Se fertilizer application 75 g ha^−1^; Se2, Se fertilizer application 150 g ha^−1^; Se3, Se fertilizer application 225 g ha^−1^; Se4, Se fertilizer application 300 g ha^−1^. Different lowercase letters in the same column indicate significant differences among different treatments (*p* < 0.05). “/” The chlorophyll content in DY milk ripening period was not measured due to period delay.

**Table 4:** Pearson correlation analysis of Se fertilizer and agronomic traits, yield, and quality of dryland maize at location DY.

	Se Fertilizer	Grain Yield Per Hectare	The Total Weight	Leaf Area Tasseling	Chlorophyll Content Tasseling	Internode Length Milk Stage 4th	Plant Height Tasseling	Ear Height Tasseling	Stem Strength Tasseling 3rd	Internode Diameter Tasseling 3rd	Hard Tissue Thickness	Crude Fiber	Soluble Sugar	Crude Protein	Crude Fat	Starch	Se Content
Se fertilizer	1																
Grain yield per hectare	−0.163	1															
The total weight	−0.350	−0.044	1														
Leaf area	0.375 *	−0.086	−0.431	1													
Chlorophyll content	0.007	0.268	0.239	−0.201	1												
Internode length	−0.060	−0.005	0.343	−0.534 *	0.568 *	1											
Plant height	−0.446	−0.116	0.253	0.015	−0.143	−0.046	1										
Ear height tasseling	−0.176	−0.337	0.337	0.005	−0.415	−0.255	−0.060	1									
stem strength	0.727 **	0.045	−0.579 *	0.597 *	0.200	0.079	−0.285	−0.502	1								
internode diameter Tasseling 3rd	0.133	0.273	−0.058	−0.004	0.725 **	0.631 *	−0.252	−0.390	0.590 *	1							
Hard tissue thickness	0.633 **	−0.169	−0.305	0.339	0.285	−0.028	−0.380	−0.400	0.668 *	0.157	1						
Crude fiber	0.831 **	−0.076	−0.353	0.552 *	0.262	−0.307	−0.434	−0.226	0.675 **	0.307	0.782 **	1					
Soluble sugar	−0.529 *	0.582 *	0.046	−0.248	0.015	−0.159	−0.125	0.012	−0.345	−0.252	−0.044	−0.138	1				
Crude protein	0.872 **	−0.339	−0.414	0.598 *	0.225	−0.289	−0.322	−0.220	0.681 **	0.364	0.830 **	0.940 **	−0.331	1			
Crude fat	−0.266	−0.388	0.291	−0.110	−0.058	−0.165	0.375	0.438	−0.427	−0.122	0.101	−0.277	0.052	−0.157	1		
Starch	0.875 **	0.032	−0.506	0.500	0.296	−0.094	−0.608 *	−0.323	0.756 **	0.468	0.834 **	0.866 **	−0.152	0.806 **	−0.546 *	1	
Se content	0.579 *	−0.130	−0.298	0.001	0.383	0.085	−0.855 **	−0.039	0.400	0.406	0.569	0.626 *	0.038	0.538 *	−0.326	0.707 **	1

Means with * and ** are significantly different at *p* < 0.05 and *p* < 0.01, respectively.

**Table 5:** Pearson correlation analysis of Se fertilizer and agronomic traits, yield, and quality of dryland maize at location SY.

	Se Fertilizer	Grain Yield Per Hectare	The Total Weight	Leaf Area Tasseling	Chlorophyll Content Tasseling	Internode Length Milk Stage 4th	Plant Height Tasseling	Ear Height Tasseling	Stem Strength Tasseling 3rd	Internode Diameter Tasseling 3rd	Hard Tissue Thickness	Crude Fiber	Soluble Sugar	Crude Protein	Crude Fat	Starch	Se Content
Se fertilizer	1																
Grain yield per hectare	−0.238	1															
The total weight	−0.140	0.812 **	1														
Leaf area	−0.139	0.215	0.418	1													
Chlorophyll content	−0.066	0.040	0.088	−0.142	1												
Internode length	−0.068	0.500 **	0.750 **	−0.065	0.255	1											
Plant height	0.389	−0.513	−0.437	−0.563 *	−0.108	−0.434	1										
Ear height tasseling	−0.135	−0.720 **	−0.556 *	−0.546 *	−0.061	−0.363	0.652 **	1									
stem strength	0.150	0.332	0.145	−0.021	0.008	0.415 *	−0.101	−0.165	1								
internode diameter tasseling 3rd	−0.148	0.365 *	0.730 **	0.087	0.130	0.800 **	−0.382	−0.377	0.327	1							
Hard tissue thickness	−0.551 *	0.800 **	0.805 **	0.351	0.065	0.661 **	−0.524	−0.438	0.356	0.672 **	1						
Crude fiber	0.585 *	−0.521 *	−0.537 *	−0.353	−0.115	−0.727 **	0.252	0.192	−0.389	−0.778 **	−0.733 **	1					
Soluble sugar	0.604 *	−0.384	−0.130	−0.277	−0.544 *	−0.346	0.087	−0.060	−0.056	−0.401	−0.454	0.412	1				
Crude protein	−0.104	0.861 **	0.665 **	0.497	0.405	0.484	−0.500	−0.820 **	0.140	0.568 *	0.416	−0.264	−0.244	1			
Crude fat	−0.116	0.603 *	0.589 *	0.532	0.183	0.492	−0.592 *	−0.650 *	0.071	0.472	0.255	−0.257	0.083	0.780 **	1		
Starch	0.688 **	−0.026	0.129	−0.112	−0.076	−0.185	−0.013	−0.355	−0.063	−0.180	−0.285	0.474	0.657 **	0.293	0.442	1	
Se content	0.963 **	−0.380	−0.305	−0.492	−0.150	−0.459	0.515	0.025	−0.085	−0.538 *	−0.638 *	0.676 **	0.574 *	−0.177	−0.242	0.665 **	1

Means with * and ** are significantly different at *p* < 0.05 and *p* < 0.01, respectively.

**Table 6:** Effects of Se fertilizer application on plant height and ear height of maize at locations DY and SY.

Site	Treatment	Internode Length (cm)			Plant Height (cm)		Ear Height (cm)
		Milk Stage 3rd	Milk Stage 4th	Milk Stage 5th	Tasseling	Milk Stage	Tasseling
DY	Se0	16.0 ± 0.8b	19.5 ± 1.4b	21.5 ± 0.5c	310.3 ± 4.5b	308.0 ± 3.5b	109.0 ± 15.7a
	Se1	13.8 ± 1.3a	17.2 ± 1.7ab	20.0 ± 1.2bc	307.0 ± 3.6ab	305.7 ± 3.1ab	118.0 ± 6.24a
	Se2	13.1 ± 1.0a	17.0 ± 1.8ab	19.9 ± 1.6bc	304.0 ± 6.8ab	299.7 ± 2.3a	107.7 ± 6.0a
	Se3	12.7 ± 1.6a	15.9 ± 1.1a	18.1 ± 0.9ab	300.2 ± 9.4a	300.7 ± 6.7ab	103.7 ± 7.1a
	Se4	13.8 ± 0.3a	15.2 ± 0.4a	17.4 ± 0.7a	306.5 ± 3.9ab	303.3 ± 3.5ab	110.3 ± 11.0a
SY	Se0	17.8 ± 4.0a	21.7 ± 1.9a	27.6 ± 2.3b	343.7 ± 9.7a	328.7 ± 4.2a	150.0 ± 2.6c
	Se1	15.2 ± 1.7a	20.3 ± 2.8a	24.5 ± 2.0a	341.2 ± 6.3a	325.3 ± 3.2a	137.0 ± 7.2ab
	Se2	16.2 ± 2.2a	20.8 ± 3.4a	24.2 ± 2.0a	345.3 ± 9.8a	325.0 ± 2.6a	130.7 ± 2.9a
	Se3	15.0 ± 1.0a	19.1 ± 2.0a	23.7 ± 1.8a	344.1 ± 6.a	330.3 ± 3.5a	140.0 ± 6.2abc
	Se4	16.6 ± 1.3a	21.3 ± 2.2a	25.7 ± 2.2ab	338.8 ± 11.6a	331.7 ± 4.5a	144.7 ± 7.2bc

Note: Se0, Se fertilizer application 0 g ha^−1^; Se1, Se fertilizer application 75 g ha^−1^; Se2, Se fertilizer application 150 g ha^−1^; Se3, Se fertilizer application 225 g ha^−1^; Se4, Se fertilizer application 300 g ha^−1^. Different lowercase letters in the same column indicate significant differences among different treatments (*p* < 0.05).

**Table 7:** Effects of Se fertilizer application on stem cortex of maize at locations DY and SY.

Site	Treatment	Hard Tissue Thickness (mm)	Cortex Thickness (μm)	Crude Fiber (%)
DY	Se0	1.2 ± 0.03a	56.9 ± 4.6ab	34.5 ± 1.0a
	Se2	1.3 ± 0.03b	61.8 ± 2.6b	37.4 ± 1.0bc
	Se3	1.3 ± 0.04b	54.7 ± 2.6a	38.4 ± 1.0c
	Se4	1.3 ± 0.04b	54.2 ± 5.0a	38.3 ± 1.0c
SY	Se0	1.3 ± 0.01b	64.2 ± 2.9a	38.2 ± 1.0bc
	Se2	1.3 ± 0.03c	60.2 ± 2.3a	38.8 ± 1.0c
	Se3	1.3 ± 0.01b	60.3 ± 2.7a	36.8 ± 1.0ab
	Se4	1.1 ± 0.03a	58.8 ± 6.2a	43.3 ± 1.0d

Note: Se0, Se fertilizer application 0 g ha^−1^; Se2, Se fertilizer application 150 g ha^−1^; Se3, Se fertilizer application 225 g ha^−1^; Se4, Se fertilizer application 300 g ha^−1^. Different lowercase letters in the same column indicate significant differences among different treatments (*p* < 0.05).

**Table 8:** Effects of Se fertilizer application on quality of maize at locations DY and SY.

Site	Treatment	Soluble Sugar (g 100 g^−1^)	Crude Protein (g 100 g^−1^)	Crude Fat (g 100 g^−1^)	Starch (g 100 g^−1^)
DY	Se0	2.3 ± 0.1b	8.3 ± 0.2a	3.5 ± 0.1b	70.3 ± 0.2a
	Se1	2.4 ± 0.1c	8.6 ± 0.2a	3.9 ± 0.1c	70.6 ± 0.2a
	Se2	2.4 ± 0.1c	8.7 ± 0.2a	3.3 ± 0.1a	72.4 ± 0.2b
	Se3	2.4 ± 0.1bc	9.3 ± 0.2b	3.5 ± 0.1b	72.9 ± 0.2c
	Se4	2.1 ± 0.1a	9.3 ± 0.2b	3.5 ± 0.1b	72.5 ± 0.2b
SY	Se0	2.6 ± 0.2a	7.9 ± 0.1a	2.9 ± 0.1a	70.9 ± 2.0a
	Se1	2.7 ± 0.02ab	8.2 ± 0.1b	3.3 ± 0.1b	73.4 ± 2.0ab
	Se2	2.5 ± 0.2a	8.5 ± 0.1c	3.2 ± 0.1b	74.5 ± 2.0ab
	Se3	2.9 ± 0.2b	8.0 ± 0.1a	3.0 ± 0.1a	75.1 ± 2.0b
	Se4	2.9 ± 0.2b	8.0 ± 0.1a	3.0 ± 0.1a	76.1 ± 2.0b

Note: Se0, Se fertilizer application 0 g ha^−1^; Se1, Se fertilizer application 75 g ha^−1^; Se2, Se fertilizer application 150 g ha^−1^; Se3, Se fertilizer application 225 g ha^−1^; Se4, Se fertilizer application 300 g ha^−1^. Different lowercase letters in the same column indicate significant differences among different treatments (*p* < 0.05).

## Data Availability

All data generated or analyzed during this study are included in this published article.

## References

[1] D’Amato R., Feudis M.D., Guiducci M., Businelli D. (2019). *Zea mays* L. Grain: Increase in Nutraceutical and Antioxidant Properties Due to Se Fortification in Low and High Water Regimes. J. Agric. Food Chem..

[2] Bocchini M., D’Amato R., Ciancaleoni S., Fontanella M.C., Palmerini C.A., Beone G.M., Onofri A., Negri V., Marconi G., Albertini E. (2018). Soil Selenium (Se) Biofortification Changes the Physiological, Biochemical and Epigenetic Responses to Water Stress in *Zea mays* L. by Inducing a Higher Drought Tolerance. Front. Plant Sci..

[3] Zilić S., Serpen A., Akıllıoğlu G., Gökmen V., Vančetović J. (2012). Phenolic compounds, carotenoids, anthocyanins and antioxidant capacity of colored maize (*Zea mays* L.) kernels. J. Agric. Food Chem..

[4] Grujcic D., Yazici A.M., Tutus Y., Cakmak I., Singh B.R. (2021). Biofortification of Silage Maize with Zinc, Iron and Selenium as Affected by Nitrogen Fertilization. Plants.

[5] Dinh Q.T., Cui Z., Huang J., Tran T.A.T., Wang D., Yang W., Zhou F., Wang M., Yu D., Liang D. (2018). Selenium distribution in the Chinese environment and its relationship with human health: A review. Environ. Int..

[6] Wang D., Zhou F., Yang W., Peng Q., Man N., Liang D. (2017). Selenate redistribution during aging in different Chinese soils and the dominant influential factors. Chemosphere.

[7] Nawaz F., Ashraf M.Y., Ahmad R., Waraich E.A., Shabbir R.N., Bukhari M.A. (2015). Supplemental selenium improves wheat grain yield and quality through alterations in biochemical processes under normal and water deficit conditions. Food Chem..

[8] Pieters A.J., El Souki S. (2005). Effects of drought during grain filling on PS II activity in rice. J. Plant Physiol..

[9] Nawaz F., Ahmad R., Waraich E.A., Naeem M.S., Shabbir R.N. (2012). Nutrient Uptake, Physiological Responses, and Yield Attributes of Wheat (*Triticum Aestivum* L.) Exposed to Early and Late Drought Stress. J. Plant Nutr..

[10] Ngigi P.B., Lachat C., Masinde P.W., Laing G.D. (2019). Agronomic biofortification of maize and beans in Kenya through selenium fertilization. Environ. Geochem. Health.

[11] Yang C., Yao H., Wu Y., Sun G., Yang W., Li Z., Shang L. (2020). Status and risks of selenium deficiency in a traditional selenium-deficient area in Northeast China. Sci. Total Environ..

[12] White P.J. (2016). Selenium accumulation by plants. Ann. Bot..

[13] Cartes P., Jara A.A., Pinilla L., Rosas A., Mora M.L. (2010). Selenium improves the antioxidant ability against aluminium-induced oxidative stress in ryegrass roots. Ann. Appl. Biol..

[14] Li J., Yang W., Guo A., Yang S., Chen J., Qiao Y., Anwar S., Wang K., Yang Z., Gao Z. (2021). Combined foliar and soil selenium fertilizer improves selenium transport and the diversity of rhizosphere bacterial community in oats. Environ. Sci. Pollut. Res. Int..

[15] Dong J.Z., Wang Y., Wang S.H., Yin L.P., Xu G.J., Zheng C., Lei C., Zhang M.Z. (2013). Selenium increases chlorogenic acid, chlorophyll and carotenoids of *Lycium chinenseleaves*. J. Sci. Food Agric..

[16] Chilimba A.D.C., Young S.D., Black C.R., Meacham M.C., Lammel J., Broadley M.R. (2012). Agronomic biofortification of maize with selenium (Se) in Malawi. Field Crops Res..

[17] Luo H.W., He L.X., Du B., Wang Z.M., Zheng A.X., Lai R.F., Tang X.R. (2019). Foliar application of selenium (Se) at heading stage induces regulation of photosynthesis, yield formation, and quality characteristics in fragrant rice. Photosynthetica.

[18] Fontanella M.C., D'Amato R., Regni L., Proietti P., Beone G.M., Businelli D. (2017). Selenium speciation profiles in biofortified sangiovese wine. J. Trace Elem. Med. Biol..

[19] Yao X., Chu J., Wang G. (2009). Effects of selenium on wheat seedlings under drought stress. Biol. Trace Elem. Res..

[20] Ghouri F., Ali Z., Naeem M., Ul-Allah S., Babar M., Baloch F.S., Chattah W.S., Shahid M.Q. (2021). Effects of Silicon and Selenium in Alleviation of Drought Stress in Rice. Silicon.

[21] Klopfenstein T.J., Erickson G.E., Berger L.L. (2013). Maize is a critically important source of food, feed, energy and forage in the USA. Field Crops Res..

[22] Ranum P., Peña-Rosas J.P., Garcia-Casal M.N. (2014). Global maize production, utilization, and consumption. Ann. N. Y. Acad. Sci..

[23] Dinh Q.T., Li Z., Tran T.A.T., Wang D., Liang D. (2017). Role of organic acids on the bioavailability of selenium in soil: A review. Chemosphere.

[24] Feudis M.D., D’Amato R., Businelli D., Guiducci M. (2019). Fate of selenium in soil: A case study in a maize (*Zea mays* L.) field under two irrigation regimes and fertilized with sodium selenite. Sci. Total Environ..

[25] Kaur N., Sharma S., Kaur S., Nayyar H. (2014). Selenium in agriculture: A nutrient or contaminant for crops?. Arch. Agron. Soil Sci..

[26] Izydorczyk G., Ligas B., Mikula K., Witek-Krowiak A., Moustakas K., Chojnacka K. (2021). Biofortification of edible plants with selenium and iodine—A systematic literature review. Sci. Total Environ..

[27] Nawaz F., Naeem M., Ashraf M.Y., Tahir M.N., Zulfiqar B., Salahuddin M., Shabbir R.N., Aslam M. (2016). Selenium Supplementation Affects Physiological and Biochemical Processes to Improve Fodder Yield and Quality of Maize (*Zea mays* L.) under Water Deficit Conditions. Front. Plant Sci..

[28] Zhang F., Li X., Wu Q., Lu P., Kang Q., Zhao M., Wang A., Dong Q., Sun M., Yang Z. (2022). Selenium Application Enhances the Accumulation of Flavones and Anthocyanins in Bread Wheat (*Triticum aestivum* L.) Grains. J. Agric. Food Chem..

[29] Liu K., Cai M., Hu C., Sun X., Cheng Q., Jia W., Yang T., Nie M., Zhao X. (2019). Selenium (Se) reduces Sclerotinia stem rot disease incidence of oilseed rape by increasing plant Se concentration and shifting soil microbial community and functional profiles. Environ. Pollut..

[30] Zhu S., Liang Y., Gao D., An X., Kong F. (2017). Spraying foliar selenium fertilizer on quality of table grape (*Vitis vinifera* L.) from different source varieties. Sci. Hortic..

[31] Halli H.M., Angadi S., Kumar A., Govindasamy P., Madar R., Baskar V.D., Elansary H.O., Tamam N., Abdelbacki A.M.M., Abdelmohsen S.A.M. (2021). Assessment of Planting Method and Deficit Irrigation Impacts on Physio-Morphology, Grain Yield and Water Use Efficiency of Maize (*Zea mays* L.) on Vertisols of Semi-Arid Tropics. Plants.

[32] Gui J.Y., Rao S., Huang X., Liu X., Cheng S., Xu F. (2022). Interaction between selenium and essential micronutrient elements in plants: A systematic review. Sci. Total Environ..

[33] Yang H., Yang X., Ning Z., Kwon S.Y., Li M.-L., Tack F.M.G., Kwon E.E., Rinklebe J., Yin R. (2022). The beneficial and hazardous effects of selenium on the health of the soil-plant-human system: An overview. J. Hazard. Mater..

[34] Poldma P., Tonutare T., Viitak A., Luik A., Moor U. (2011). Effect of selenium treatment on mineral nutrition, bulb size, and antioxidant properties of garlic (*Allium sativum* L.). J. Agric. Food Chem..

[35] Luo H., Xing P., Liu J., Pan S., Tang X., Duan M. (2021). Selenium improved antioxidant response and photosynthesis in fragrant rice (*Oryza sativa* L.) seedlings during drought stress. Physiol. Mol. Biol. Plants.

[36] Taha R.S., Seleiman M.F., Shami A., Alhammad B.A., Mahdi A.H.A. (2021). Integrated Application of Selenium and Silicon Enhances Growth and Anatomical Structure, Antioxidant Defense System and Yield of Wheat Grown in Salt-Stressed Soil. Plants.

[37] Xie L., Wen D., Wu C., Zhang C. (2022). Transcriptome analysis reveals the mechanism of internode development affecting maize stalk strength. BMC Plant Biol..

[38] Li X., Sun J., Li W., Gong Z., Jia C., Li P. (2022). Effect of foliar application of the selenium-rich nutrient solution on the selenium accumulation in grains of Foxtail millet (Zhangzagu 10). Environ. Sci. Pollut. Res. Int..

[39] Wang S., Liang D., Wang D., Wei W., Fu D., Lin Z. (2012). Selenium fractionation and speciation in agriculture soils and accumulation in corn (*Zea mays* L.) under field conditions in Shaanxi Province, China. Sci. Total Environ..

[40] Netto A.T., Campostrini E., de Oliveira J.G., Bressan-Smith R.E. (2005). Photosynthetic pigments, nitrogen, chlorophyll a fluorescence and SPAD-502 readings in coffee leaves. Sci. Hortic..

[41] Zhang S., Zhao G., Lang K., Su B., Chen X., Xi X., Zhang H. (2019). Integrated Satellite, Unmanned Aerial Vehicle (UAV) and Ground Inversion of the SPAD of Winter Wheat in the Reviving Stage. Sensors.

[42] Saavedra T., Gama F., Rodrigues M.A., Abadia J., de Varennes A., Pestana M., Da Silva J.P., Correia P.J. (2022). Effects of foliar application of organic acids on strawberry plants. Plant Physiol. Biochem..

[43] Li J., Yang W., Guo A., Qi Z., Chen J., Huang T., Yang Z., Gao Z., Sun M., Wang J. (2021). Combined foliar and soil selenium fertilizer increased the grain yield, quality, total se, and organic Se content in naked oats. J. Cereal Sci..

